# A new section: neuroimaging through clinical cases

**DOI:** 10.1590/1980-57642015DN940000317

**Published:** 2015

**Authors:** Ricardo Nitrini

**Affiliations:** Editor-in-Chief

In view of the advances in neuroimaging, it is very important for health providers involved in caring for subjects with cognitive disorders to have a basic knowledge of imaging in their curriculum. Today, neuroimaging is an essential part of the complementary investigation in cognitive and behavioral neurology and neuropsychiatry. Imaging allows inferences to be drawn on which brain regions or cognitive networks are affected, while new developments in imaging biomarkers enable the assessment of perfusion, metabolism and protein deposition patterns. Thus, neuroimaging is a new area of interface between clinicians and radiologists. However, as for all fields of knowledge, it is sometimes difficult to stay abreast, especially in fields undergoing constant development. With these premises, we are introducing a new section in *Dementia & Neuropsychologia* entitled *"Neuroimaging through clinical cases"* focusing on short case reports (maximum of 750 words, without abstract, no more than 10 references) with interesting imaging features. This will cover not only rare imaging cases, but also include typical or emblematic imaging features of several diseases along with cognitive repercussions and biomarker images in healthy and pathological states. The section will also focus on teaching neuroimaging through discussion of clinical cases and help readers to become familiarized with neuroimaging. The editors of this new section are Leandro Tavares Lucato and Fabio Henrique de Gobbi Porto. We began this section with a case of Korsakoff syndrome that presented as a rapidly progressive dementia with contrast enhancement in both mammillary bodies on magnetic resonance imaging, which proved essential to confirm the etiology. We hope this section will inform readers interested in learning neuroimaging and widen the spectrum of the journal.

**Ricardo Nitrini** Editor-in-Chief

